# PM_2.5_ in Beijing – temporal pattern and its association with influenza

**DOI:** 10.1186/1476-069X-13-102

**Published:** 2014-12-04

**Authors:** Yijia Liang, Liqun Fang, Hui Pan, Kezhong Zhang, Haidong Kan, Jeffrey R Brook, Qinghua Sun

**Affiliations:** Division of Environmental Health Sciences, College of Public Health, The Ohio State University, 424 Cunz Hall, 1841 Neil Avenue, Columbus, OH 43210 USA; Upper Arlington High School, Upper Arlington, OH USA; Beijing Institute of Microbiology and Epidemiology, Beijing, China; Peking Union Medical College Hospital, Beijing, China; Center for Molecular Medicine & Genetics, Wayne State University School of Medicine, Detroit, MI USA; School of Public Health, Key Lab of Public Health Safety of the Ministry of Education, & Key Lab of Health Technology Assessment of the Ministry of Health, Fudan University, Shanghai, China; Research Institute for the Changing Global Environment and Fudan Tyndall Centre, Fudan University, Shanghai, China; Shanghai Key Laboratory of Atmospheric Particle Pollution and Prevention, Fudan University, Shanghai, China; Environment Canada, Toronto, Canada; Shanghai Key Laboratory of Meteorology and Health, Shanghai, China

**Keywords:** Air pollution, PM_2.5_, Temporal pattern, Association, Influenza, Beijing

## Abstract

**Background:**

Air pollution in Beijing, especially PM_2.5,_ has received increasing attention in the past years. Despite Beijing being one of the most polluted cities in the world, there has still been a lack of quantitative research regarding the health impact of PM_2.5_ on the impact of diseases in Beijing. In this study, we aimed to characterize temporal pattern of PM_2.5_ and its potential association with human influenza in Beijing.

**Methods:**

Based on the data collected on hourly ambient PM_2.5_ from year 2008 to 2013 and on monthly human influenza cases from 2008 and 2011, we investigated temporal patterns of PM_2.5_ over the five-year period and utilized the wavelet approach to exploring the potential association between PM_2.5_ and influenza.

**Results:**

Our results found that ambient PM_2.5_ pollution was severe in Beijing with PM_2.5_ concentrations being significantly higher than the standards of the World Health Organization, the US EPA, and the Chinese EPA in the majority of days during the study period. Furthermore, PM_2.5_ concentrations in the winter heating seasons were higher than those in non-heating seasons despite high variations. We also found significant association between ambient PM_2.5_ peak and human influenza case increase with a delayed effect (e.g. delayed effect of PM_2.5_ on influenza).

**Conclusions:**

Ambient PM_2.5_ concentrations were significantly associated with human influenza cases in Beijing, which have important implications for public health and environmental actions.

## Introduction

Air pollution has long been held as one of the foremost threats to human health, especially in metropolitan areas throughout the world [1–4]. In China, rapid urbanization has been recognized as a cause of increased amounts of air pollution, especially in the capital city of Beijing [5]. Some reports indicated that air pollution in Beijing to be worsening every year, especially for PM_2.5_, with concentrations vastly exceeding China’s urban hourly standard of 75 μg/m^3^ [6–9]. The numbers of migrants and automobiles in Beijing are set to continue as the Chinese government looks to urbanization to grow its economy, and thus PM_2.5_ pollution is becoming ever important to study as detrimental to the health of the residents, especially those living in big metropolitan areas globally [10] (Table 1).

**Table 1:** **Characteristics of PM**_**2.5**_ **measurements and days exceeding the standards by the SEPA, EPA, and the WHO from 2008 to 2013**

Year	Measurements (days)	Range (lowest, highest)	Annual mean	Days ^1^(%)	Days ^2^(%)	Days ^3^(%)
2008	212	(5.8 235.38)	84.74 (50.31)	105 (49.53)	174 (82.08)	194 (91.51)
2009	263	(13.94, 482.25)	104.65(70.41)	165 (62.74)	227 (86.31)	239 (90.87)
2010	320	(9.0, 441.5)	104.89 (76.38)	186 (58.13)	267 (83.44)	286 (89.38)
2011	333	(10.38, 492.75)	98.30 (79.65)	170 (51.05)	257 (77.18)	279 (83.78)
2012	329	(2.92, 428.54)	91.74 (68.85)	169 (51.37)	246 (74.77)	278 (84.50)
2013	289	(10.5, 568.57)	103.04 (83.77)	149 (51.56)	242 (83.74)	262 (90.66)

Days^1^ – days with 24-hr average exceeding the SEPA standard; days^2^ – days with 24-hr average exceeding the EPA standard; days^3^ – days with 24-hr average exceeding the WHO standard.

PM_2.5,_ particles with an aerodynamic diameter of less than 2.5 μm, has some important chemical and physical characteristics, varying from place to place [11]. Many chemical constituents, such as sulfates, nitrates, ammonium, inorganic ions, organic elemental carbon, particle-bound water, polycyclic aromatic hydrocarbons (PAH), and among other, can be part of the composition of PM_2.5_ [12]. In Beijing, carbonaceous aerosols and major ions (sulfate, nitrate and ammonium) constituted more than 69% of PM_2.5_ with the major sources coming from dust, secondary sulfate and nitrate, coal combustion, and diesel and gasoline exhaust [8]. Exposure to PM_2.5_ has already been linked to many health issues, such as arrhythmia [13], and systemic vascular dysfunction [14], among others. For the millions living and moving to Beijing, daily inhalation of polluted air could become a major health issue, with the government already advising citizens to stay inside on high concentration days [15]. Therefore, the potential risk for increased mortality rates merits increased research in PM_2.5_ pollution itself, but despite PM_2.5_ pollution becoming a prevalent problem, the public, especially the ordinary residents in China lack of access to the quality and quantity of air quality data. Occasionally, the data come in small intervals, with one study only obtaining access to 37 days [16]. Unfortunately, small sample sizes do not allow for thorough understanding of the problem at hand. Moreover, air pollution has become a long-term problem as well; many health effects are chronic due to long-term exposure. As such, it becomes important to analyze longer trends of air pollution in order to establish a clearer understanding of what the future might bring.

In addition to these health effects associated with PM_2.5_ exposure, there is increasing concern over its association with infectious diseases, some of which have caused major global health issues. Of particular note is influenza, which is a major global health problem, especially in China [17, 18]. Geographic variations in influenza in China are prevalent throughout the nation, creating complex problems regarding vaccination programs [19]. Special strains of influenza outbreaks in China have presented challenges of adequately addressing influenza health crises [20]. Influenza complications can lead to severe respiratory diseases, a health issue that has also been linked with PM_2.5_ exposure [21]. Though ostensibly different health issues, exposure to PM_2.5_ can potentially exacerbate flu symptoms, yet no study has explored the relationship between PM_2.5_ exposure and influenza cases. To address this, we studied the measurements collected from Embassy of the United States in Beijing, ranging from 2008 to 2013, obtaining detailed information regarding PM_2.5_ concentrations on an hourly basis, and influenza case data from Beijing in an attempt to establish such a relationship, which may contribute to the future changes in environmental regulation and public health policies.

## Materials and methods

### Sources of data

Hourly measurements of PM_2.5_ from April 2008 to October 2013 were collected from an air quality monitoring site at the US Embassy in Beijing. The Embassy used two US EPA approved air quality monitors [MetOne BAM 1020] to measure PM_2.5_ particulates at hourly intervals in the Chaoyang district, where the Embassy compound is located (39.953°N 116.459°E). We chose the Embassy’s air pollution data because it recorded detailed measurements of PM_2.5_ over a long period of time, despite originating from only one source. Other sources of PM_2.5_ data include Chinese government agencies, but their released data are sporadic (short-term and discontinuous), limiting proper characterization of PM_2.5_ [16]. The hourly measurements (C_hr_) were converted to daily average  and monthly average  for subsequent analyses described below. From December 2008 to February 2009, the Embassy air quality monitor had malfunctions, leading to missing measurements for this three-month period. In addition to describing characteristics of PM_2.5_ concentrations over the five-year period, we were also interested in potential relationships between air quality and health impacts, specifically respiratory infections. In this study, we explored the association between PM_2.5_ concentrations and human influenza in urban area of Beijing. In China, influenza has been on the list of Class C notifiable diseases as part of China’s national infectious diseases surveillance (now called National Infectious Diseases Reporting Systems, or NIDR) since 1970 and individual cases were reported by hospitals and clinics required by law [20]. At participating surveillance hospitals, each influenza-like case (defined as the one with body temperature ≥38°C and cough or sore throat) is recruited, sample (nasal or throat swab) is taken and sent for laboratory confirmation. Sample handling and processing follow national standard protocol by Ministry of Health [22]. Both influenza-like case and confirmed case information are then reported to the NIDR system [22]. Nevertheless, under-reporting does exist, as elaborated further in discussion section. In this study, reported influenza case data from January 2008 to December 2011 for all 17 districts in Beijing were retrieved from the National Infectious Disease Reporting system (NIDR). The reported cases from the NIDR system were aggregated for each district at the monthly interval. Since the US Embassy is located in Chaoyang district, reflecting a typical downtown setting of Beijing, we assume that the PM_2.5_ measurements from the US Embassy were likely representative of downtown environment. To explore the possible association between the PM_2.5_ and human influenza, we aggregated human influenza cases for the following eight districts which include and/or surround downtown areas: Haidian, Dongcheng, Xicheng, Chaoyang, Fengtai, Shijingshan, Chongwen, and Xuanwu districts. The aggregated data and PM_2.5_ measurements were then explored using the Wavelet analysis described below.

### Descriptive and wavelet analyses

Descriptive statistical analyses were performed to characterize the following aspects of air quality in Beijing: daily and monthly averages, minimum and maximum concentrations of PM_2.5_, peak days/seasons of PM_2.5_, days of exceeding the standard for PM_2.5_ by the World Health Organization [23], the US EPA and the Chinese EPA [6],, monthly variations in PM_2.5_ concentrations by overlaying the six years’ monthly averages.

Like many environmental and epidemiological time-series data, the PM_2.5_ and influenza time-series data are subject to non-stationary properties which are usually complex [24]. To account for these factors, we used wavelet analysis to characterize the temporal patterns of monthly PM_2.5_ concentrations, following the general approach described by Cazelles *et al* [24]. Wavelet analysis was used to decompose a time-series from a time domain to time-frequency (or time-period) domain and detect localized intermittent periods of a time-series [25]. Following the general approach described by Grinsted *et al.* [26] we used cross wavelet coherence approach to explore the possible association between the two time-series, PM_2.5_concentrations and human influenza cases. The cross wavelet angle was used to assess the phase difference between components of the two time series (for technical details, please refer to (Grinsted *et al.*, 2004)). A Matlab package for performing cross wavelet coherence analysis was downloaded for the analysis (http://noc.ac.uk/using-science/crosswavelet-wavelet-coherence).

## Results

### Descriptive analysis

**Figure 1:**
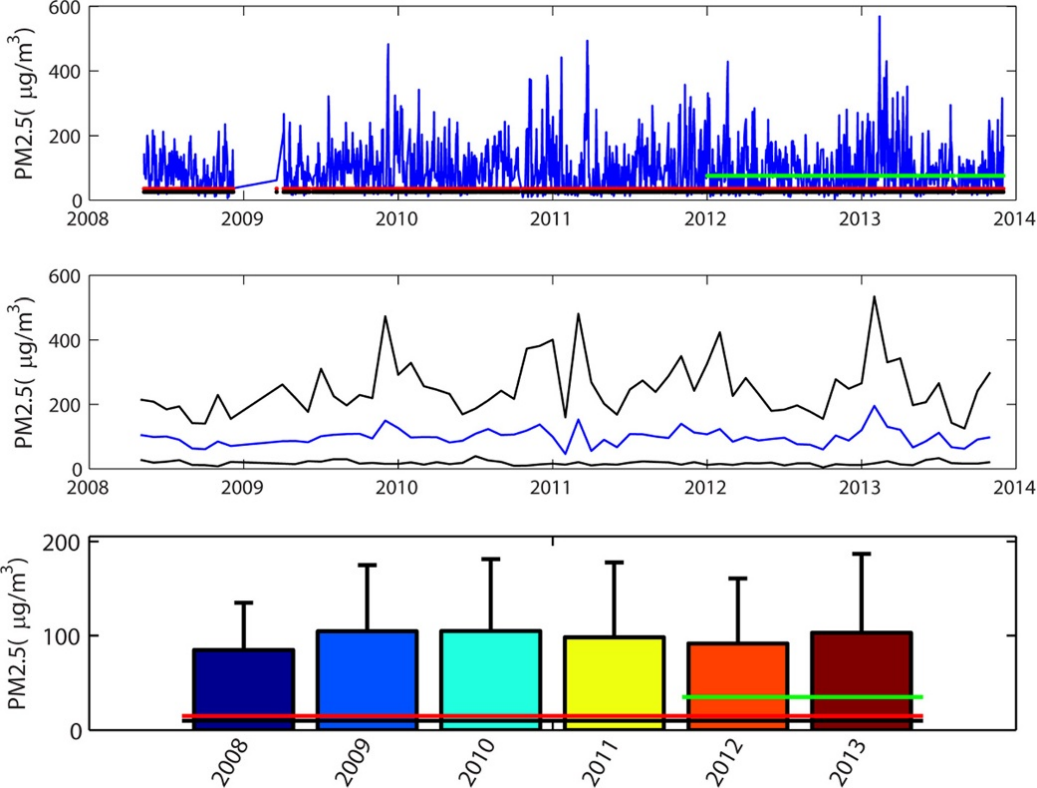
**PM**_**2.5**_ **concentrations (μg/m** ^**3**^ **) from 2008 to 2013 in Beijing.** Top-24-hour averages of PM_2.5_ measurements superimposed with standards by the WHO (25 μg/m^3^ for the 24-hour average, black line), the USEPA (35 μg/m^3^ for the 24-hour average, redline), and the Chinese State Environmental Protection Administration (SEPA, 75 μg/m^3^, implemented from 2012, green line; the 75 μg/m^3^ standard, or Class II standard, was developed specific for residential, commercial, industrial, and heavy-traffic areas) (ref, Cao et al. *Chinese and US air quality standard evolution paper)*. Middle - Monthly variations in average, 97.5 and 2.5 percentiles of PM_2.5_ from 2008 to 2013 in Beijing. Bottom – Annual averages of PM_2.5_ concentrations from 2008 to 2013, superimposed with the standards by the WHO (10 μg/m^3^ for the annual average, black line), the US EPA (15 μg/m^3^ for the annual average, redline, the EPA standard was reduced to 12 μg/m^3^ after 2013), and the SEPA (35 μg/m^3^, implemented from 2012).

**Figure 2:**
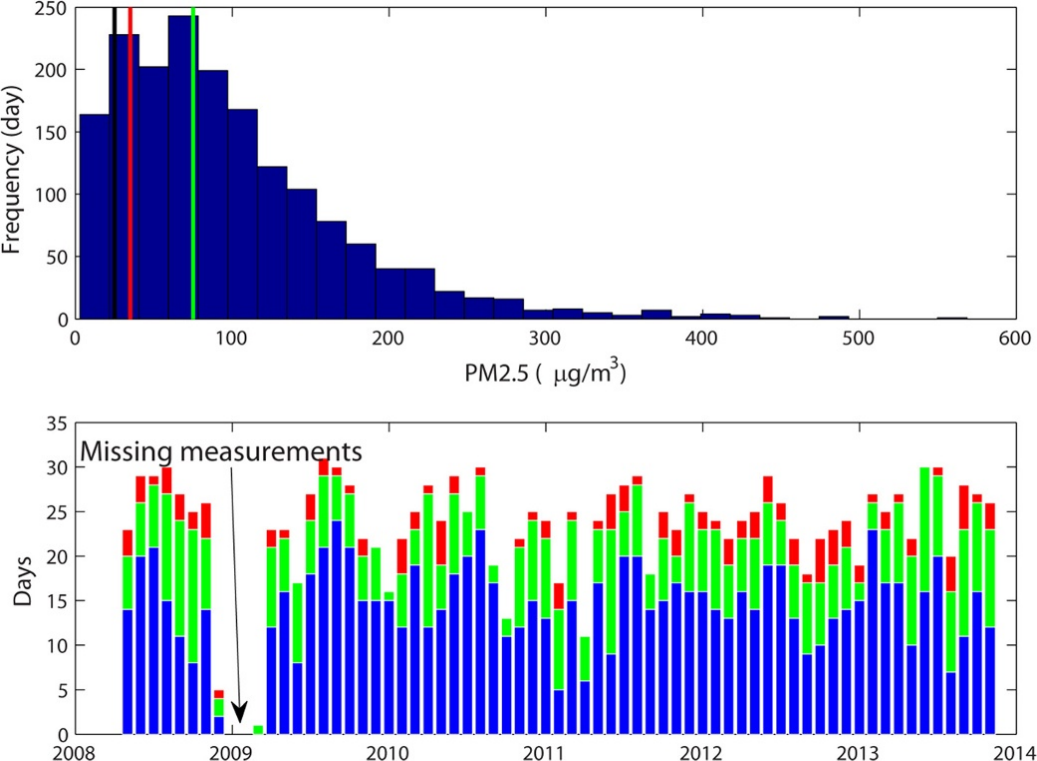
**Distribution of PM**_**2.5**_ **concentrations and days with 24-hr averages exceeding the standards.** Top – frequency distribution of days with varying 24-hr average concentrations for the whole study period; black line – the WHO standard (25 μg/m^3^), redline – the EPA standard (35 μg/m^3^), green – the SEPA standard (75 μg/m^3^). Bottom – distribution of days each month with the 24-hr averages exceeding the standards by the WHO (red), the EPA (green) and SEPA (blue). The gap between December 2008 and February 2009 was due to missing measurements.

**Figure 3:**
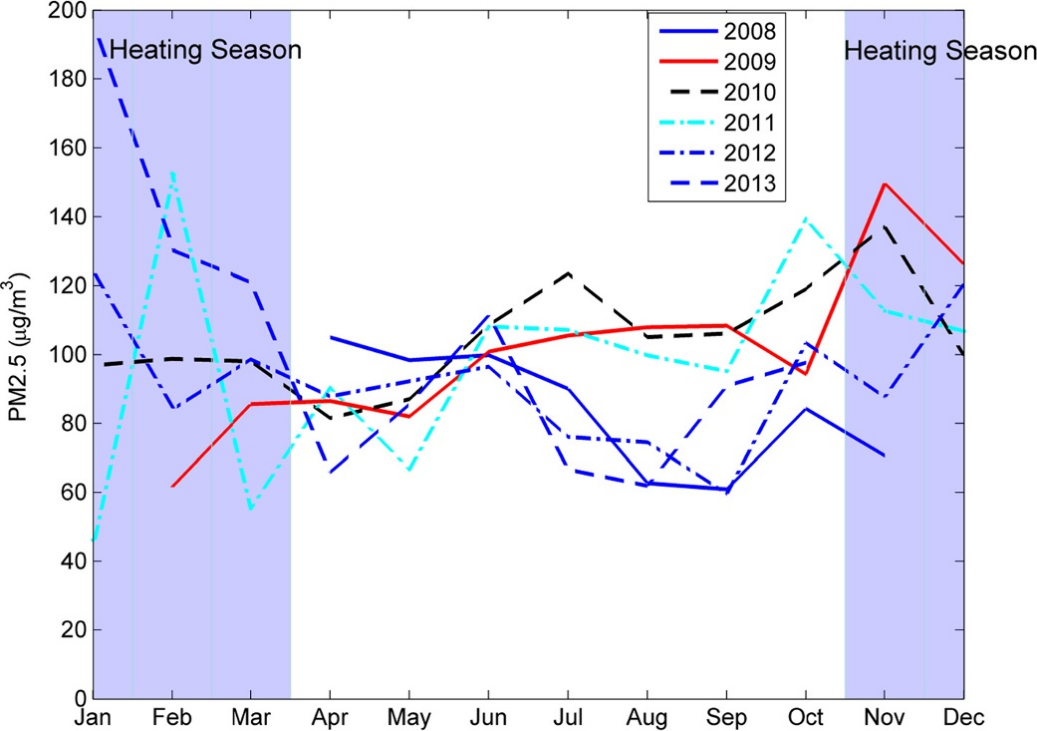
**Monthly variations in PM**_**2.5**_ **in Beijing for each year between 2008 and 2013.** The light blue shaded area covers from the mid-November to mid-March, typically winter season in Beijing during which centralized heating is provided. Overall, the PM_2.5_ concentrations are higher in the winter season. During 2008, the year of the Beijing Olympics, the Chinese government imposed regulatory controls on emissions, which may be a mechanistic explanation for why concentrations in 2008 are generally lower in each month than concentrations in other years.

The PM_2.5_ measurements covered a span of five years, from 2008 to 2013, encompassing 43,255 hourly measurements. Figure 1 shows the hourly measurements of PM_2.5_ over the five-year period and indicates that, for the most part, PM_2.5_ concentrations way exceed the US EPA and Chinese standards of 35 μg/m^3^. Figure 1 also shows the monthly average, 97.5 and 2.5 percentiles of monthly measurements, superimposed with the 35 μg/m^3^ line showing the US EPA and the Chinese National standard. Clearly, all monthly averages exceeded the standard (Figure 1). Overall, the PM_2.5_ concentrations were lowest in 2008 and then increased in the following years (Figure 1). We counted days for each month with daily average over the standard for the whole study period and Figure 2 shows the distribution of days with the daily PM_2.5_ exceeding the national standard (Figure 2). Only 328 days out of 1746 days met the standard and the remaining 1418 days (the number of days exceeding the 35 μg/m^3^ standard was 81.21%) exceeding the standard with the range between more than 35 μg/m^3^ to 550 μg/m^3^ (Figure 2). Note that there was a three-month period during which the measurements were missing due to malfunctions of the equipment. Figure 3 shows monthly averages over a span of 12 months superimposed for each year from 2008-13, in order to identify potential season trends. From April to June, the monthly averages are similar for every year, but besides that period there is quite a bit of variation. There are clear within- and between-year variations in monthly averages of PM_2.5_ – overall, the months with lowest concentrations were clustered between April and May, and highest months were concentrated between October and December with the highest variations in January (Figure 3). The heating season in Beijing, which starts in mid-November and ends in mid-March, is shaded in blue. Generally, PM_2.5_ concentrations tend to rise during this period (Figure 3).

### Wavelet analysis

**Figure 4:**
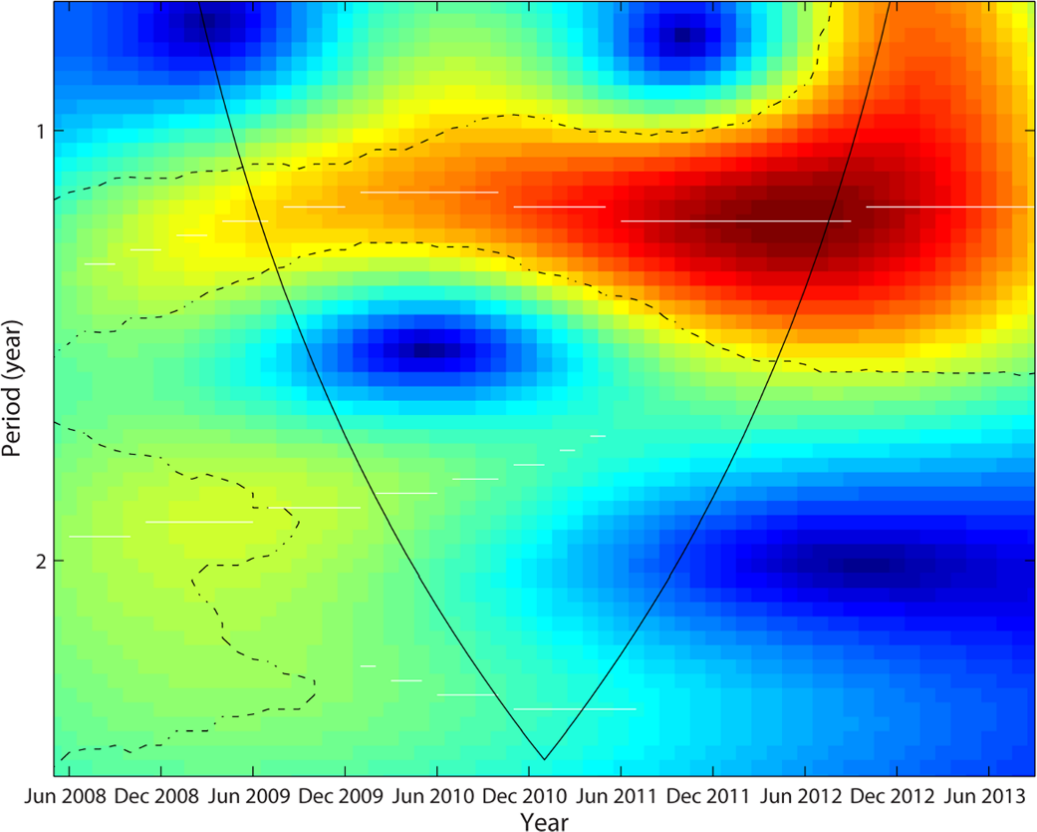
**Wavelet power spectrum analysis of monthly PM**_**2.5**_ **concentrations from year 2008 to 2013 in Beijing.** The color code for power values from dark blue (low value) to dark red (high value). The dotted white lines show the maxima of the undulations of the wavelet power spectrum and the dotted-dashed show the α = 5% significant levels and the cone of influence which indicates the region not influenced by edge (following the algorithm/software by Cazelle et al. 2007).

To explore the evolution of the oscillating characteristics of the time-series of PM_2.5_ at the monthly interval over the five-year period in Beijing, we used the wavelet approach. Transient dynamic relationships were observed as shown in Figure 4 – the monthly PM_2.5_ over the study period was dominated by a slightly greater than 1-year mode, particularly after 2009. During the 2008 and 2009, weak dominant modes (1-1.3 years) were observed (Figure 4).

**Figure 5:**
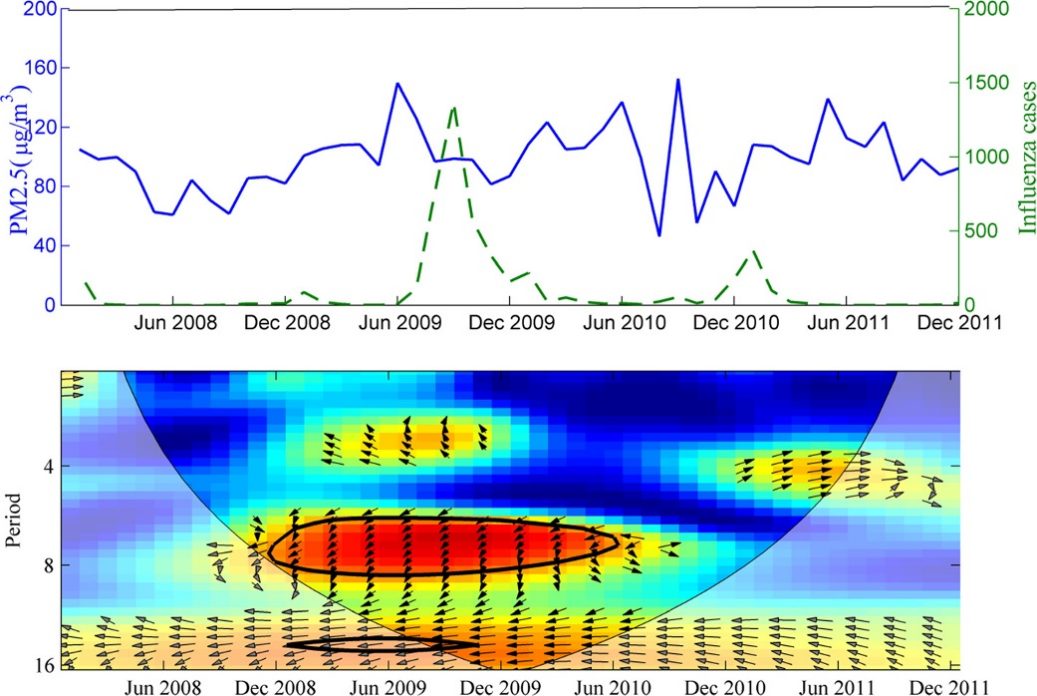
**The association between monthly PM**_**2.5**_ **(μg/m** ^**3**^ **) and reported influenza cases (in five urban districts) from 2008 to 2011 in Beijing.** Top – monthly PM_2.5_ and reported influenza cases. Bottom: Wavelet coherence between the standardized PM_2.5_ measurements and reported influenza cases in Beijing. The 5% significance level against red noise is shown as a thick contour. The relative phase relationship is shown as arrows, suggesting that PM_2.5_ leads influenza by about 90^o^ pointing down, equivalent to 1-2 months delay in occurrence.

We also use the cross wavelet approach to explore the potential relationship between PM_2.5_ concentrations and human influenza. Figure 5 shows the time profiles of PM_2.5_ concentrations and human influenza reported in urban areas of Beijing from 2008 to 2011 (Figure 5). Interestingly, the wavelet coherence analysis shows that there is clearly significant correlation between the two quantities from December 2008 to June 2010 period and downward phase angle (close to 90°, equivalent to 1-2 months), suggesting that there was a delay in the occurrence of influenza following PM_2.5_ (Figure 5).

## Discussion

Many studies, in particular epidemiological studies, have demonstrated strong evidence for the association between particulate air pollution (from both PM_10_ and PM_2.5_) and human illness, in particular cardiovascular and respiratory disease. Both short- and long-term exposures to ambient particulate matters have implications for different health impacts [13, 27–31]. In China, much attention on air pollution has been focused on PM_10_, and the studies about PM_2.5_ exposure on health impact in China have just started. In this study, we report a five-year period of PM_2.5_ measurements from an urban site of Beijing and its possible association with human influenza. To our knowledge, this is the first study reporting comprehensive measurements of PM_2.5_ related to influenza in Beijing, China.

Although the present study only used data from one monitoring site in Beijing, it certainly reflects the unacceptably high air pollution occurring in one of the most populated cities in the world. From the monitoring records, on average, more than 81% of days each year, people live in the environment with polluted air exceeding US EPA and Chinese health standards. PM_2.5_ has increasingly been shown to be more harmful to human health than bigger particles since the smaller particles have more potential to be deposited in the alveoli and even penetrate the blood-gas barrier [32]. However, China has only been recently releasing PM_2.5_ concentrations to the public in major cities. We all recognize that significant efforts have been made, but record-breaking pollution levels were recorded in the winter of 2013 in Beijing, China [33]. This indicates that more comprehensive efforts still are needed so that the air quality improvements during the Beijing Olympic Games in 2008 could be sustainable [34, 35].

Air pollution has been implicated in respiratory illness infections [36]. Moreover, many reports are related to indoor air pollution (e.g. combustion) which has been related to acute lower respiratory infections [37–40] and this has been a concern in many developing countries. There are not many studies related to ambient air pollution and infections, although there are reports on association between air pollution and pneumococcal disease related to respiratory viruses [41]. In this study, we chose to explore the potential linkage between exposure to PM_2.5_ and human influenza, given the consideration of nature of the disease, biological plausibility, and availability of the influenza data. Using three-year’s data, our study suggested that human influenza cases were correlated to PM_2.5_ concentrations in Beijing and the finding is in general agreement with other studies [42]. This seems to have a time lag while peaks of PM_2.5_ levels were followed by peaks of influenza in 2009 and 2010. Interestingly, the peak of PM_2.5_ levels were not obvious in 2008, so was relatively moderate for influenza cases during the same year, which further strengthens the correlation between PM_2.5_ exposure and influenza occurrence. The underlying possible mechanisms related to this are complex. There are some reports about ambient air pollution on respiratory infections in humans [43, 44] and it is likely that air pollution exposure causes host defense disorders, including both innate and cell-mediated immune responses against bacterial and viral infections [45, 46]. Various experimental studies have suggested that the deposition of particulates on epithelial cells that line the airways activates inflammatory signaling cascades [47, 48]. In addition, high PM_2.5_ levels could precipitate inflammatory and tissue remodeling changes in the lungs [49].

The study is also subject to some limitations. First, we only used one measurement source from the US Embassy, limiting spatial representativeness of the present work. Second, our data suffer from under-reporting of influenza cases; however, the under-reporting tends to occur in consistent pattern throughout the country and we believe that the relative pattern (e.g. seasonal variations in influenza) still holds. Third, the observational time periods of five years for PM_2.5_ levels and three years for influenza cases are relatively short, especially when a definite, strong correlation is concluded. Fourth, one kind of infectious disease, such as influenza in our study, may not be ideal, and more similar respiratory infectious diseases should be included in the future to demonstrate strong and broad correlation between PM_2.5_ and infectious diseases. Nevertheless, this study demonstrates a temporal correlation between PM_2.5_ pollution and influenza peak occurrence in Beijing, which provides significant impact on both environmental policy-making and public health preparedness.

In conclusion, despite the limitations, our study has indicated severity of PM_2.5_ pollution in urban Beijing and health implications associated with human influenza. Further studies are in urgent need to understand the mechanisms underlying the potential association as well as public health and environmental policy implications.

## Abbreviations

PM_2.5_: Fine particulate matters
US: The United States of America
US EPA: United States Environmental Protection Agency.

## References

[1] Mehta S, Shin H, Burnett R, North T, Cohen AJ (2013). Ambient particulate air pollution and acute lower respiratory infections: a systematic review and implications for estimating the global burden of disease. Air Qual Atmos Health.

[2] Cohen AJ, Ross Anderson H, Ostro B, Pandey KD, Krzyzanowski M, Kunzli N, Gutschmidt K, Pope A, Romieu I, Samet JM, Smith K (2005). The global burden of disease due to outdoor air pollution. J Toxic Environ Health A.

[3] Cohen AJ (2000). Outdoor air pollution and lung cancer. Environ Health Perspect.

[4] Cohen AJ, Pope CA (1995). Lung cancer and air pollution. Environ Health Perspect.

[5] Tuo Y, Li X, Wang JC (2013). Negative effects of Beijing’s air pollution caused by urbanization on residents’ health.

[6] Cao JJ, Chow JC, Lee FSC, Watson JG (2013). Evolution of PM2.5 measurements and standards in the US and future perspectives for China. Aerosol Air Qual Res.

[7] Han LH, Zhuang GS, Cheng SY, Wang HY (2009). Characteristics of re-suspended road dust and its significant effect on the airborne particulate pollution in Beijing. Huan Jing Ke Xue = Huanjing Kexue / [Bian Ji, Zhongguo Ke Xue Yuan Huan Jing Ke Xue Wei Yuan Hui "Huan Jing Ke Xue" Bian Ji Wei Yuan Hui].

[8] Liu Z, Hu B, Wang L, Wu F, Gao W, Wang Y (2014). Seasonal and diurnal variation in particulate matter (PM and PM) at an urban site of Beijing: analyses from a 9-year study. Environ Sci Pollut Res Int.

[9] Zhang M, Song Y, Cai X (2007). A health-based assessment of particulate air pollution in urban areas of Beijing in 2000-2004. Sci Total Environ.

[10] Gong P, Liang S, Carlton EJ, Jiang Q, Wu J, Wang L, Remais JV (2012). Urbanisation and health in China. Lancet.

[11] Schlesinger RB (2007). The health impact of common inorganic components of fine particulate matter (PM2.5) in ambient air: a critical review. Inhal Toxicol.

[12] WHO (2013). Health effects of particulate matter: policy implications for countries in Eastern Europe, Caucasus and Central Asia.

[13] Brunekreef B, Holgate ST (2002). Air pollution and health. Lancet.

[14] Kampfrath T, Maiseyeu A, Ying Z, Shah Z, Deiuliis JA, Xu X, Kherada N, Brook RD, Reddy KM, Padture NP, Parthasarathy S, Chen LC, Moffatt-Bruce S, Sun Q, Morawietz H, Rajagopalan S (2011). Chronic fine particulate matter exposure induces systemic vascular dysfunction via NADPH oxidase and TLR4 pathways. Circ Res.

[15] **Contigency plans of severe air pollution of Beijing City** In *(2012) #34*. China; 2013. http://www.bjmemc.com.cn/g327/s921/t1866.aspx

[16] Zhang A, Qi Q, Jiang L, Zhou F, Wang J (2013). Population exposure to PM2.5 in the urban area of Beijing. PLoS One.

[17] Jiang W, Liu S, Hou G, Li J, Zhuang Q, Wang S, Zhang P, Chen J (2012). Chinese and global distribution of H9 subtype avian influenza viruses. PLoS One.

[18] Moscona A (2009). Global transmission of oseltamivir-resistant influenza. N Engl J Med.

[19] Yu H, Alonso WJ, Feng L, Tan Y, Shu Y, Yang W, Viboud C (2013). Characterization of regional influenza seasonality patterns in China and implications for vaccination strategies: spatio-temporal modeling of surveillance data. PLoS Med.

[20] Wang L, Wang Y, Jin S, Wu Z, Chin DP, Koplan JP, Wilson ME (2008). Emergence and control of infectious diseases in China. Lancet.

[21] Qiu H, Yu IT, Tian L, Wang X, Tse LA, Tam W, Wong TW (2012). Effects of coarse particulate matter on emergency hospital admissions for respiratory diseases: a time-series analysis in Hong Kong. Environ Health Perspect.

[22] MoH (2010). Protocol for national influenza surveillance.

[23] WHO (2013). Health effects of particulate matter.

[24] Cazelles B, Chavez M, Magny GC, Guegan JF, Hales S (2007). Time-dependent spectral analysis of epidemiological time-series with wavelets. J R Soc Interface.

[25] Torrence C, Compo GP (1998). A practical guide to wavelet analysis. Bull Am Meteorol Soc.

[26] Grinsted A, Moore JC, Jevrejeva S (2004). Application of the cross wavelet transform and wavelet coherence to geophysical time series. Nonlinear Process Geophys.

[27] Brunekreef B (2007). Health effects of air pollution observed in cohort studies in Europe. J Expo Sci Environ Epidemiol.

[28] Brunekreef B (2011). Air pollution and health–air quality standards. Verh K Acad Geneeskd Belg.

[29] Roemer W, Hoek G, Brunekreef B (1993). Effect of ambient winter air pollution on respiratory health of children with chronic respiratory symptoms. Am Rev Respir Dis.

[30] van der Zee S, Hoek G, Boezen HM, Schouten JP, van Wijnen JH, Brunekreef B (1999). Acute effects of urban air pollution on respiratory health of children with and without chronic respiratory symptoms. Occup Environ Med.

[31] van der Zee SC, Hoek G, Boezen MH, Schouten JP, van Wijnen JH, Brunekreef B (2000). Acute effects of air pollution on respiratory health of 50-70 yr old adults. Eur Respir J.

[32] Sun Q, Hong X, Wold LE (2010). Cardiovascular effects of ambient particulate air pollution exposure. Circulation.

[33] Steins R (2013). China’s air pollution linked to millions of early deaths.

[34] Rich DQ, Kipen HM, Huang W, Wang GF, Wang YD, Zhu P, Ohman-Strickland P, Hu M, Philipp C, Diehl SR, Lu SE, Tong J, Gong J, Thomas D, Zhu T, Zhang JJ (2012). Association between changes in air pollution levels during the Beijing olympics and biomarkers of inflammation and thrombosis in healthy young adults. Jama J Am Med Assoc.

[35] Xu X, Deng F, Guo X, Lv P, Zhong M, Liu C, Wang A, Tzan K, Jiang SY, Lippmann M, Rajagopalan S, Qu Q, Chen LC, Sun Q (2012). Association of systemic inflammation with marked changes in particulate air pollution in Beijing in 2008. Toxicol Lett.

[36] Chauhan AJ, Johnston SL (2003). Air pollution and infection in respiratory illness. Br Med Bull.

[37] Ezzati M, Kammen D (2001). Indoor air pollution from biomass combustion and acute respiratory infections in Kenya: an exposure-response study. Lancet.

[38] Pandey MR, Boleij JS, Smith KR, Wafula EM (1989). Indoor air pollution in developing countries and acute respiratory infection in children. Lancet.

[39] Smith KR (2000). National burden of disease in India from indoor air pollution. Proc Natl Acad Sci U S A.

[40] Smith KR, Samet JM, Romieu I, Bruce N (2000). Indoor air pollution in developing countries and acute lower respiratory infections in children. Thorax.

[41] Kim PE, Musher DM, Glezen WP, Rodriguez-Barradas MC, Nahm WK, Wright CE (1996). Association of invasive pneumococcal disease with season, atmospheric conditions, air pollution, and the isolation of respiratory viruses. Clin Infect Dis.

[42] Wong CM, Yang L, Thach TQ, Chau PY, Chan KP, Thomas GN, Lam TH, Wong TW, Hedley AJ, Peiris JS (2009). Modification by influenza on health effects of air pollution in Hong Kong. Environ Health Perspect.

[43] Arbex MA, Pereira LA, Carvalho-Oliveira R, Saldiva PH, Braga AL (2014). The effect of air pollution on pneumonia-related emergency department visits in a region of extensive sugar cane plantations: a 30-month time-series study. J Epidemiol Community Health.

[44] Brugha R, Grigg J (2014). Urban air pollution and respiratory infections. Paediatr Respir Rev.

[45] Xie Y, Zhang X, Tian Z, Jiang R, Chen R, Song W, Zhao J (2013). Preexposure to PM2.5 exacerbates acute viral myocarditis associated with Th17 cell. Int J Cardiol.

[46] Yin XJ, Dong CC, Ma JY, Antonini JM, Roberts JR, Barger MW, Ma JK (2005). Sustained effect of inhaled diesel exhaust particles on T-lymphocyte-mediated immune responses against *Listeria monocytogenes*. Toxicol Sci.

[47] Jin C, Shelburne CP, Li GJ, Potts EN, Riebe KJ, Sempowski GD, Foster WM, Abraham SN (2011). Particulate allergens potentiate allergic asthma in mice through sustained IgE-mediated mast cell activation. J Clin Investig.

[48] Li N, Harkema JR, Lewandowski RP, Wang MY, Bramble LA, Gookin GR, Ning Z, Kleinman MT, Sioutas C, Nel AE (2010). Ambient ultrafine particles provide a strong adjuvant effect in the secondary immune response: implication for traffic-related asthma flares. Am J Physiol Lung C.

[49] Pinkerton KE, Green FH, Saiki C, Vallyathan V, Plopper CG, Gopal V, Hung D, Bahne EB, Lin SS, Menache MG, Schenker MB (2000). Distribution of particulate matter and tissue remodeling in the human lung. Environ Health Perspect.

